# Complex Wound Closure Following Mysterious and Vicious Animal Attack

**DOI:** 10.7759/cureus.7758

**Published:** 2020-04-21

**Authors:** Karleigh R Curfman, Russell Dumire, Kamran Shayesteh

**Affiliations:** 1 Surgery, Conemaugh Memorial Medical Center, Johnstown, USA; 2 Surgery, Conemaugh Memorial Medical Center, Johnstown , USA

**Keywords:** acute trauma care, plastic and reconstructive surgery, skin graft, dermal regeneration template, animal attack, wound healing

## Abstract

Animal attacks are a worrisome and dangerous entity that occur at high volumes and are evaluated frequently by ER physicians, primary care physicians, trauma teams, acute care surgeons, and plastic surgeons. The severity of animal attacks can range from a small insect sting to mauling by large animal, and even death. With animal attacks of high intensity, there is often significant scratching, tearing, shearing, with destruction of the skin, subcutaneous tissues, muscles, and bone. Serious attacks frequently lead to infection, sepsis, pain, loss of sensation or mobility, operative interventions, and amputations of affected limbs. We report herein the traumatic mauling of a woman by a reported unknown animal. Though the entity of animal attacks has been reported in the past, this case dictates presentation given the suspicious circumstances surrounding the attack, the involvement of her care requiring a multidisciplinary surgical approach via trauma surgery and plastic surgery, multiple extensive interventions, and the excellent take of the split thickness skin graft (STSG) after the use of a dermal regeneration template (DRT).

## Introduction

Animal bites are a dangerous entity that come with serious health risks and costs, as well as injuries resulting in pain, tissue damage, and disability often requiring frequent and multiple interventions, perhaps ending in severe infection and death [1]. Attacks are reported in various forms, including: bites, stings, scratches, pecks, mauls, tramples, falls, thrown from, crushes, or gore [1]. From this variety of modalities, assaults by canines are the most common, with a reported nearly four and one half million dog bites occurring annually in the United States [2]. Approximately 900 000 people annually are treated in EDs for noncanine injuries, primarily from cats, arachnids, bees, or unknown species, where cat bites account for 400 000 of these attacks [2-3]. Given the high frequency of attacks as well as significant health concerns and difficult management associated with animal wounds, we report a patient who presented following an attack by an unknown species. She sustained major injuries to the left upper and lower extremities, face, back, shoulder, and ear with recognizable skin loss at the sites, as well as significant subcutaneous tissue and muscle damage to her extremities. These injuries necessitated a multi-disciplinary approach with multiple treatments and procedures, ultimately requiring placement of a dermal regeneration template (DRT) and subsequent split thickness skin grafting (STSG) with excellent functional result.

## Case presentation

**Figure 1:**
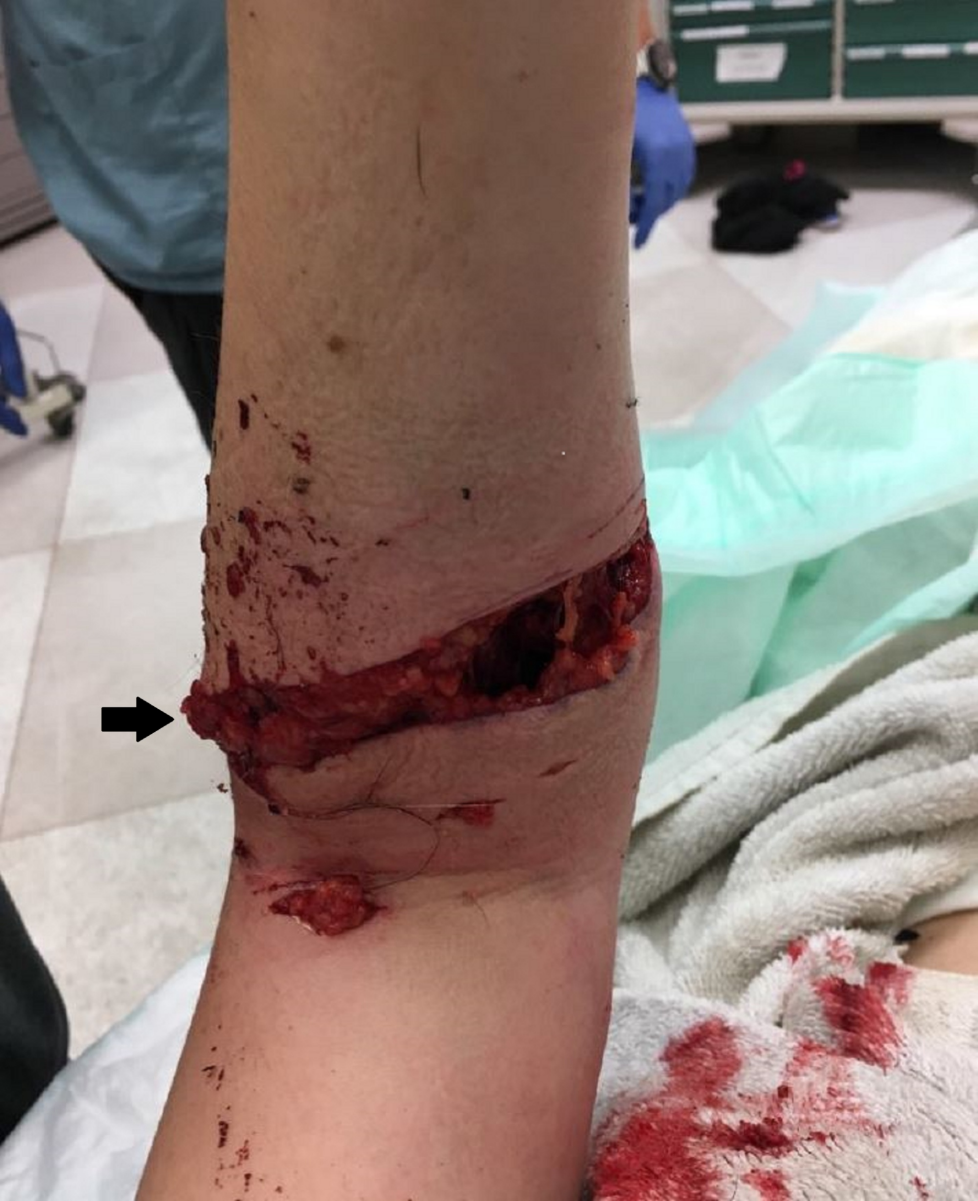
Near circumferential wound distal to left elbow from animal attack. Imaging taken in the ED upon arrival which displays a deep, over half circumferential laceration beginning medially (black arrow) and extending laterally, just distal to the patient’s left elbow.

**Figure 2:**
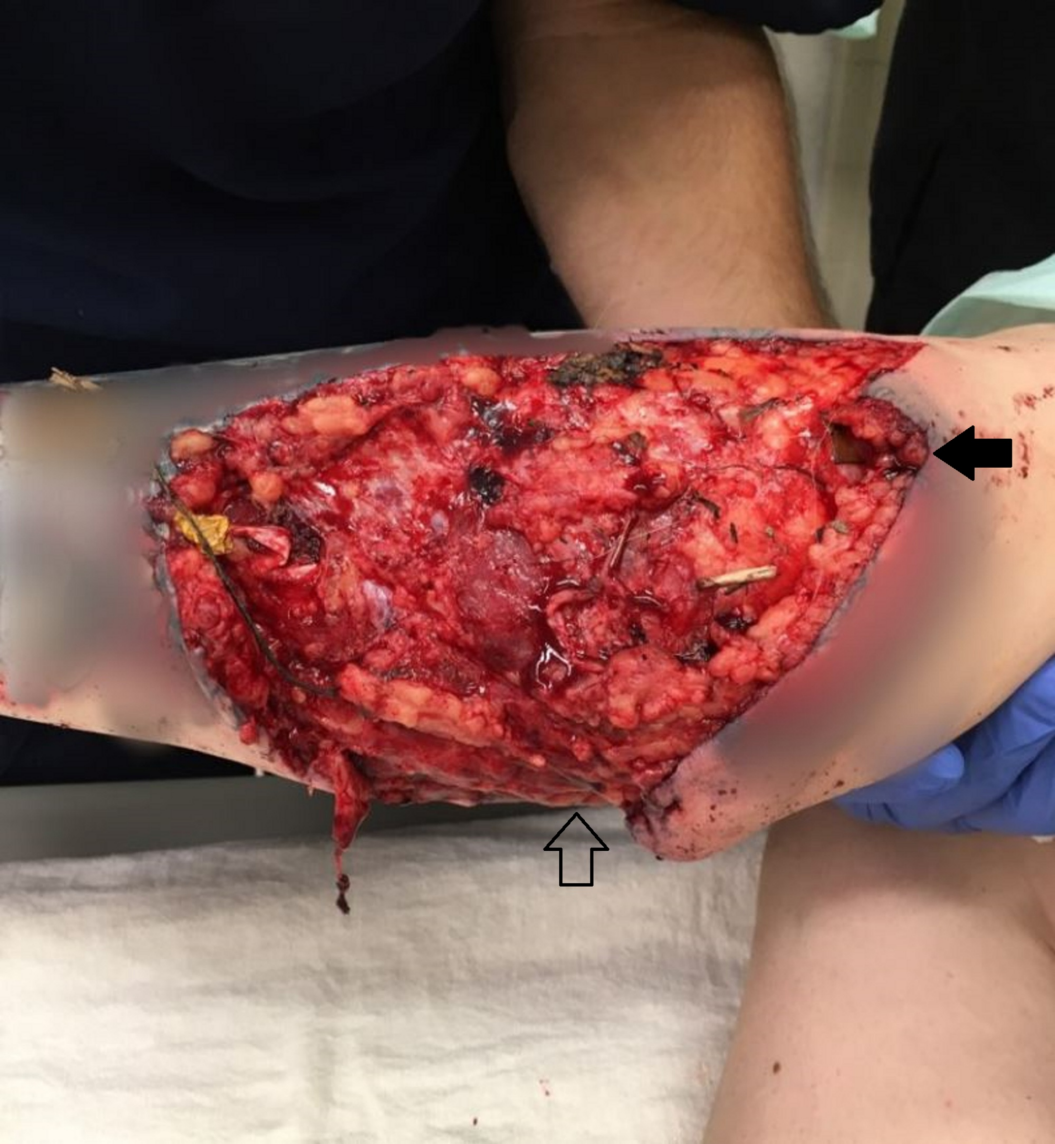
Obvious destruction of the patient's lateral lower leg. ED imaging following patient's animal attack displaying the degree of substantial damage from her attack as well as contamination of her wound. The solid arrow demarcates the patient's proximal lower leg, just distal to the knee while the outlined arrow identifies the lateral aspect of the patient's leg.

**Figure 3:**
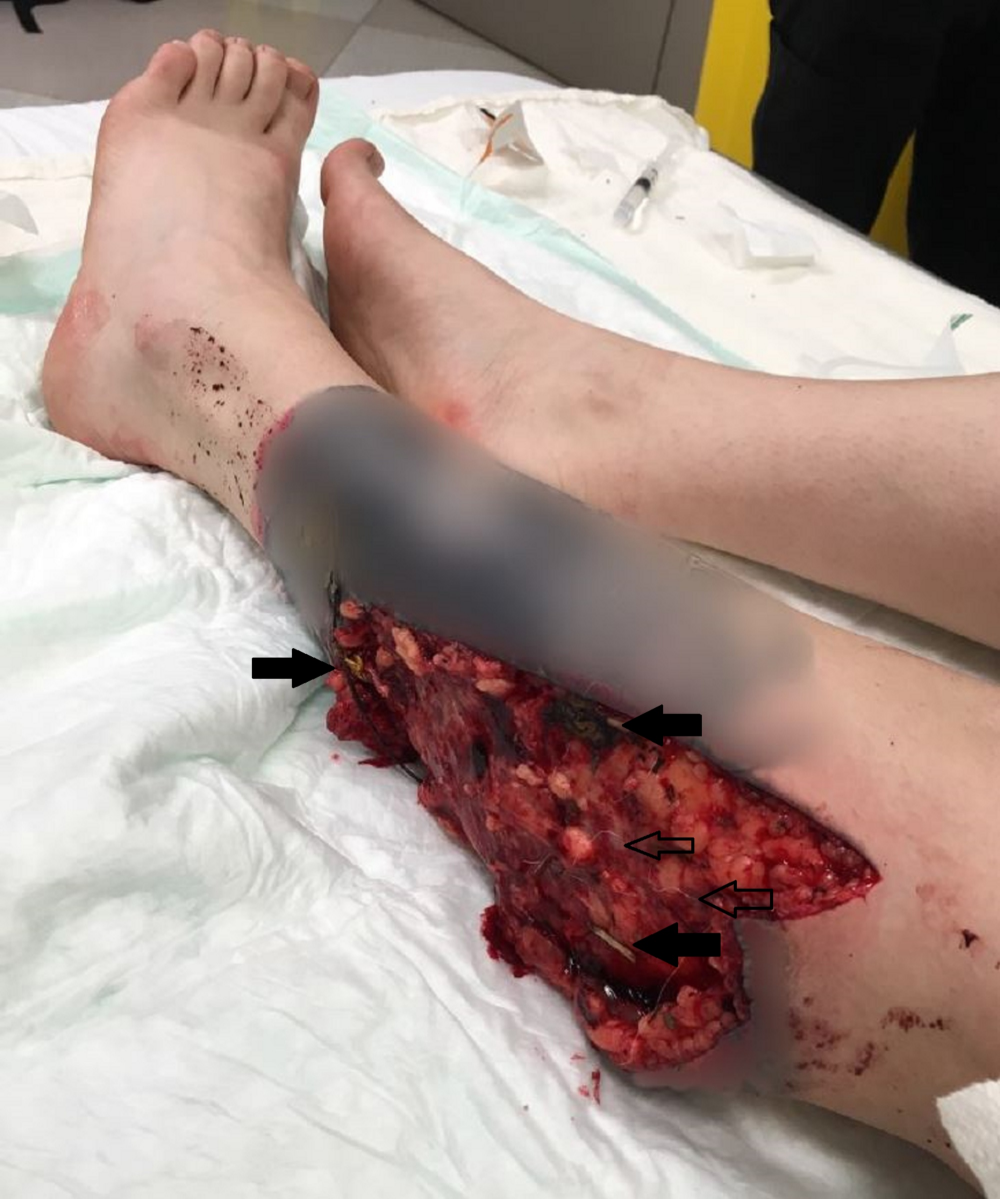
Imaging of the patient's left lower lateral leg upon arrival to initial operation. This imaging above not only displays the extent of damage to the patients lower lateral leg, but also highlights the contamination with debris from her surroundings (solid arrows) as well as attacking animal hair (outlined arrows).

**Figure 4:**
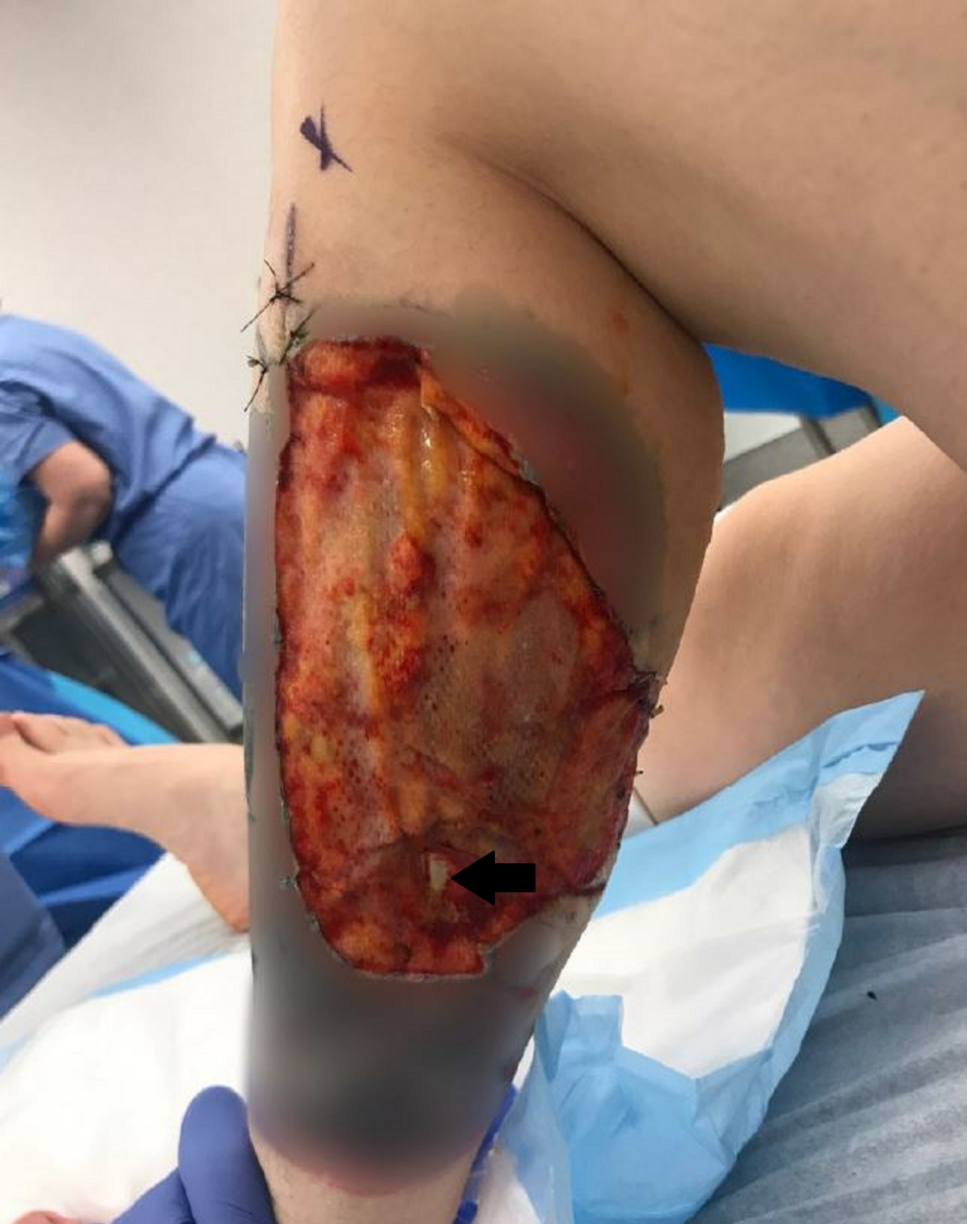
Near perfect take of the patient’s DRT. Display of an intraoperative view after the removal of the silicone layer of the DRT, two weeks after initial placement. A small, approximately 0.5 cm aspect of the template over the tendon in the central portion of the distal wound did not take (demarcated by solid black arrow), but otherwise DRT shows a near perfect response. DRT, dermal regeneration template

**Figure 5:**
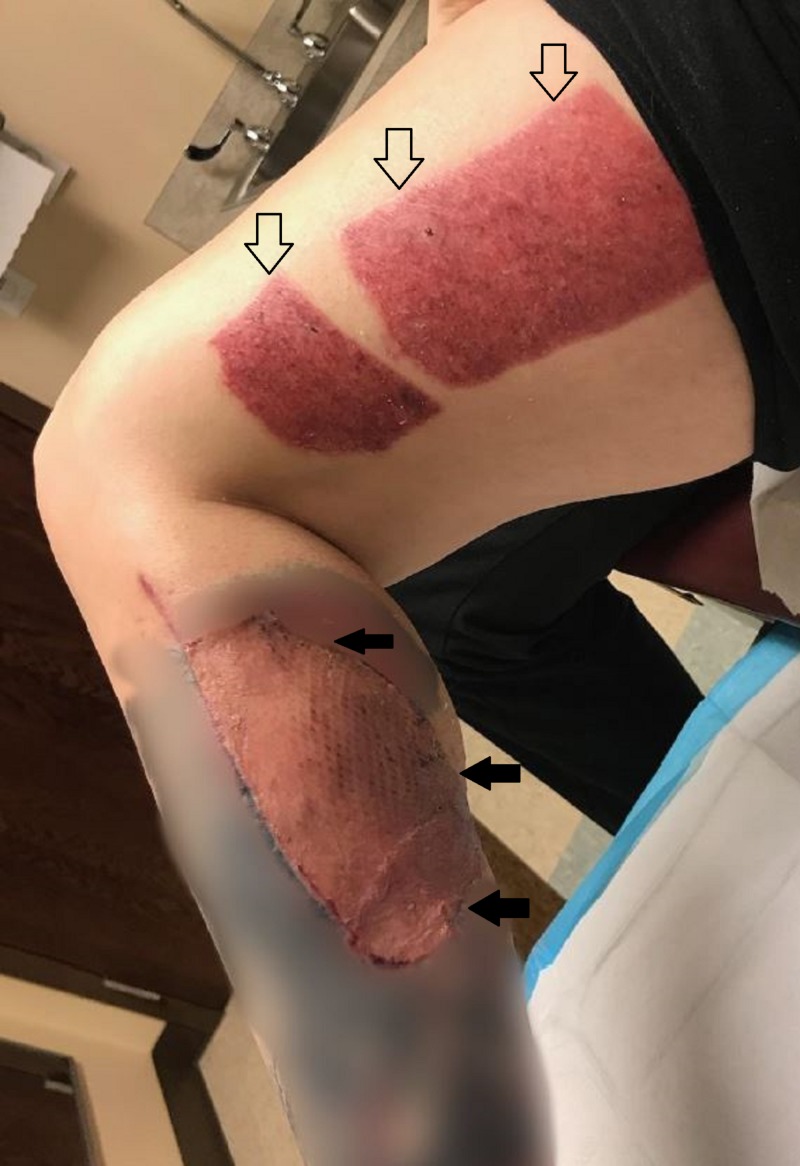
STSG and donor site six weeks after surgery. Representation of both the patient’s STSG to her lateral left lower leg (solid arrows) and the donor site on her left lateral thigh (outlined arrows). The STSG showed 100% take and no signs of infection. The patient’s donor site displays good healing and appropriate appearance and color for time. STSG, split thickness skin graft

The patient is a 30-year-old female, who presented to the ED in extreme pain after walking in the woods and being attacked by an unknown animal. There were significant wounds to her left leg, forearm, shoulder, back, ear, and face with substantial skin, subcutaneous tissue, and muscle damage. She received a tetanus booster, antibiotics, rabies vaccine, and imaging which revealed no fractures. After evaluation by the trauma team, given the nature, size, and contamination of her wounds, it was felt that emergent multi-disciplinary intervention with plastic surgery was indicated. All wounds underwent pulse lavage and were injected with rabies immunoglobulin. The wounds of the back, ear, shoulder, and face were then covered with bacitracin. Her left arm wound, with over half circumferential skin and subcutaneous tissue destruction by one deep, long laceration into the forearm musculature, measured 15 cm high and 4 cm deep (Figure 1). This was lavaged and debrided, then underwent complex closure. Her leg wound, measuring 15 cm in height and 12 cm in width, had extensive skin, subcutaneous tissues, and muscle involvement, including gastrocnemius, fibularis longus, and soleus (Figures 2-3). She miraculously had no obvious sign of vascular injury in any wound, and had full range of motion with intact sensation throughout. She continued on antibiotics, compressive wraps were placed, and her leg was splinted to prevent contracture. She returned to the OR on postoperative day (POD) four, where her lower extremity was irrigated with betadine and a bilayer DRT was placed. On POD 16, she returned to the OR where her DRT showed excellent take (98%), and no signs of infection, seroma, or hematoma (Figure 4). A meshed STSG was created and stapled into place. She was seen regularly in the outpatient setting for several months, where her STSG was noted to have 100% take, and her donor site showed appropriate healing (Figure 5).

## Discussion

Dilemma is incurred when the attacking assailant is unknown, for which management protocols have little data, which are vague and broad. Several aspects need to be considered when managing animal attack wounds including: risk for infection, closure options, cosmesis, depth, and location of injury [4]. Certain attacking species with common flora that are considered “high-risk” for infections include: cats, humans, livestock, and monkeys [4]. Bites that are also considered “high-risk” consist of those that are heavily contaminated, occur in patients with a history of diabetes mellitus, those in patients that are immunocompromised, and dog bites that are greater than eight hours old or involving the muscles, tendons, or joints [4]. Therefore, though literature has shown improved cosmesis in wounds repaired with debridement, irrigation, povidone-iodine cleaning, antibiotics, and primary closure, due to infection risk bites occurring in those situations deemed “high-risk” should not be closed, and often warrant surgical evaluation and intravenous antibiotics [4]. Prophylactic antibiotic use in animal attacks remains controversial. According to one study referenced, the only evidence-based benefit for prophylactic antibiotic use was for bites to the hand, in which infection rates were significantly decreased with use [4]. Other recommendations have shown that antibiotics can be used in “high-risk” attacks, for which amoxicillin-clavulanate is the first line therapy for prophylactic management, or azithromycin in pregnant patients, as well as a tetanus vaccination if indicated [4]. In contrast to the prior study, another study used prophylactic cephalosporins in all -68 of their participating patients, of which -26 patients showed clinical signs of infection, though only three patients had a confirmed bacteriologic source [5]. However, given the contamination and severity of attack wounds, antibiotics were recommended as first line coverage for all patients in the study [5]. Rabies vaccination may also be indicated in certain individuals. The World Health Organization (WHO) recommends two different immunization strategies, which includes post-exposure prophylaxis (PEP) and pre-exposure prophylaxis (PrEP) [6]. The WHO also categorizes exposures as Category 1 (touching or feeding of animals, licking of intact skin), Category 2 (nibbling of uncovered skin, minor scratches or abrasions without bleeding), and Category 3 (single or multiple transdermal bites or scratches, contamination of mucous membranes or broken skin with saliva from animal licks, exposure due to direct contact with bats) [6]. PEP recommendations vary based on the category of exposure: Category 1, no PEP is required, Category 2, immediate vaccination is recommended, and Category 3, immediate vaccination is recommended, as well as administration of rabies immunoglobulins (RIG), if indicated [6]. RIG is not indicated for Category 3 exposures in individuals who were previously vaccinated twice against rabies or previous PrEP completion [6]. For vaccine administration, after the wound has been adequately washed, the rabies vaccine is given immediately and continues on a multi-day schedule via intradermal or intramuscular injections, where the intradermal route in either the deltoid or thigh is favored [6]. Whereas in contrast, RIG is injected as a onetime dose directly into the wound and surrounding skin as soon as possible after PEP is initiated [6]. Conversely, PrEP is recommended for individuals at high risk of rabies exposure, including: populations in high endemic settings, occupational risk, and travelers with increased exposure risk [6]. The PrEP regimen consists of a two site intradermal vaccine injection on days zero and seven, or a one site intramuscular vaccine injection on days zero and seven [6]. 

The DRT is a development approved within the past few decades; though established, it has still yet to be accepted and implemented on the reconstructive ladder (RL) [7]. Early RLs included primary closure, skin graft, local flaps and distant flaps, and have since included microvascular repair and tissue expansion [7]. DRT is placed over the wound to promote vascularization, allowing for better quality, elasticity, and thickness of skin layers. The development has drastically affected wound coverage as DRT can be placed over burns, bones, tendons, cartilage, and historically difficult locations, like hands, feet, joints, and scalp [7]. The authors support a newly proposed RL reform, which included the addition of closure by secondary intention, negative pressure therapy, dermal templates, and free flaps [7].

Our patient’s wounds heavily influenced decision making for DRT with STSG rather than skin grafting alone or use of negative pressure vacuum assisted wound closure devices, for two reasons. Given the injury and consideration for infection, immediate autologous graft placement was deferred due to potential graft loss and donor site morbidity. DRT was chosen to both safely cover the wound, and allow for dermal regeneration to decrease the wound size for future grafting. Unlike negative pressure therapy, DRTs function by encouraging patient’s own fibroblasts to generate a neodermis formed of collagen matrix [8]. This allows the DRT to provide significant advantages, including immediate tissue wound coverage and protection from invasion, decrease in painful wound care after grafting, use of thinner autografts allowing for decreased donor site healing time, and less hypertrophic scarring [8]. However, similar to skin grafting, concern lies in DRT placement in a potentially infected field such as our own; as neodermis is unable to form in the setting of infection [8]. Conversely, one of the main benefits of negative pressure vacuum assisted wound closure devices is that they can safely be placed, and are regularly used, in infected wound settings [9]. Though, DRT is not typically recommended for placement in infected wounds, as described in our case above and a handful of sources in literature, DRTs have been placed in concerning wounds after serial debridements, irrigations, and wound monitoring where risk for potential infection has drastically decreased. Other reported cases of potential infection where DRTs have been placed include a few cases of necrotizing fasciitis, but otherwise has scarcely been reported in the literature [8, 10-11]. Understandably the cost of these technologies must be of concern and can sometimes limit treatment management. One study compared the cost of DRT to free and pedicle flaps in scalp wound coverage [12]. From this data, it was found that in smaller wounds (less than 100 cm^2^) there was no statistically significant difference in cost; however, for wounds larger than 100 cm^2^, DRTs were proven to be more cost effective when compared to flap coverage [12]. Though our patient's DRT was placed on her leg, and not her scalp such as the study, the area of her wound was nearly double the reference point of 100 cm^2^, and therefore not only was there likely clinical benefit from DRT coverage, but possibly also financial benefit.

## Conclusions

Attacks by animals are frequent and have known potential for significant injury to the patient, both physically and mentally. We feel it is important to treat every attack with diligence for strict wound assessments, irrigations, and debridement if needed. Treatment should acknowledge lavage, potential antibiotic use, and additional reconstructive surgery cautiously using DRT and/or grafting if necessary in order to achieve a clean, healthy, wound to help prevent any additional trauma, morbidities, or deterioration to the patient.
